# Matching Registered Nurse Services With Changing Care Demands in Psychiatric Hospitals: Protocol for a Multicenter Observational Study (Match^RN^ Psychiatry Study)

**DOI:** 10.2196/26700

**Published:** 2021-08-17

**Authors:** Beatrice Gehri, Stefanie Bachnick, René Schwendimann, Michael Simon

**Affiliations:** 1 Institute of Nursing Science University of Basel Basel Switzerland; 2 Department of Psychiatry University of Basel Basel Switzerland; 3 Department of Nursing Science University of Applied Sciences (hsg Bochum) Bochum Germany; 4 University Hospital Basel Basel Switzerland; 5 Nursing Research Unit Inselspital Bern University Hospital Bern Switzerland

**Keywords:** quality of care, psychiatric hospitals, nurses, patient routine data, work environment, Switzerland

## Abstract

**Background:**

The quality of care is often poorly assessed in mental health settings, and accurate evaluation requires the monitoring and comparison of not only the outcomes but also the structures and processes. The resulting data allow hospital administrators to compare their patient outcome data against those reported nationally. As Swiss psychiatric hospitals are planned and coordinated at the cantonal level, they vary considerably. In addition, nursing care structures and processes, such as nurse staffing, are only reported and aggregated at the national level, whereas nurse outcomes, such as job satisfaction or intention to leave, have yet to be assessed in Swiss psychiatric hospitals. Because they lack these key figures, psychiatric hospitals’ quality of care cannot be reasonably described.

**Objective:**

This study’s purpose is to describe health care quality by exploring hospital structures such as nurse staffing and the work environment; processes such as the rationing of care; nurse outcomes, including job satisfaction and work-life balance; and patients’ symptom burden.

**Methods:**

Match^RN^ Psychiatry is a multicenter observational study of Swiss psychiatric hospitals. The sample for this study included approximately 1300 nurses from 113 units of 13 psychiatric hospitals in Switzerland’s German-speaking region. In addition, routine patient assessment data from each participating hospital were included. The nurse survey consisted of 164 items covering three dimensions—work environment, patient safety climate, and the rationing of care. The unit-level questionnaire included 57 items, including the number of beds, number of nurses, and nurses’ education levels. Routine patient data included items such as main diagnosis, the number and duration of freedom-restrictive measures, and symptom burden at admission and discharge. Data were collected between September 2019 and June 2021. The data will be analyzed descriptively by using multilevel regression linear mixed models and generalized linear mixed models to explore associations between variables of interest.

**Results:**

The response rate from the nurse survey was 71.49% (1209/1691). All data are currently being checked for consistency and plausibility. The Match^RN^ Psychiatry study is funded by the participating psychiatric hospitals and the Swiss Psychiatric Nursing Leaders Association (Vereinigung Pflegekader Psychiatrie Schweiz).

**Conclusions:**

For the first time, the Match^RN^ Psychiatry study will systematically evaluate the quality of care in psychiatric hospitals in Switzerland in terms of organizational structures, processes, and patient and nurse outcomes. The participating psychiatric hospitals will benefit from findings that are relevant to the future planning of nurse staffing. The findings of this study will contribute to improvement strategies for nurses’ work environments and patient experiences in Swiss psychiatric hospitals.

**International Registered Report Identifier (IRRID):**

DERR1-10.2196/26700

## Introduction

### Background

As of 2016, approximately 1 billion people worldwide were affected by mental illness [1]. Although mental illnesses account for 7% of the global disease burden [2] and about 13% of total health expenditures in European Union countries [3], methods for assessing the quality of mental health care are considerably less advanced than in other health services areas [4].

We define quality of care as a measure of how fully the provided services lead to the desired outcomes [5]. Measurements of the quality of care cover various dimensions, such as structures; processes of care; and outcomes, including clinicians’ and patients’ perspectives [6,7]. To monitor the quality of care, measurements must be relevant for patients, health care providers, and policy makers and have an acceptable reporting burden [8]. On the basis of the structure-process-outcome (SPO) model by Donabedian [6], the International Psychiatric Association has concluded that differences in structures and processes are insufficiently assessed or otherwise considered in psychiatric settings. In addition, systematically monitored patient outcomes are scarce [9]. In mental health care, because research regarding structures, processes, and outcomes is limited, the quality of care in psychiatric hospitals is inadequately depicted, leaving triggers for quality improvement efforts absent.

In Switzerland, the structures of psychiatric care are planned at the cantonal level. As a result, the structures and processes of psychiatric hospitals vary considerably [10], offering the possibility to assess the impact of various structures and processes on outcomes. However, little is known about psychiatric hospitals’ nursing care structures, processes, or outcomes. For example, no requirements stipulate the number of nurses or skill or grade mix per unit, and no data are required regarding, for example, nurse well-being or job satisfaction. In contrast, patient outcomes and characteristics are well monitored. This imbalance provides a rare opportunity to examine how various structures and processes affect patient and nurse outcomes in psychiatric hospitals at the national level. By assessing them, we hope to help improve the interpretation of the mandatorily measured and reported patient outcomes. Specifically, we will provide and analyze data at the unit level, which is crucial for monitoring and describing the quality of care [11,12].

### Swiss Psychiatric Hospitals: Structures and Processes

In 2018, the 50 participating psychiatric hospitals housed 7772 beds [13] and registered 76,097 patient admissions [14], with an average stay length of 3.4 days [15]. Although the raw figures regarding the health professionals employed for 2018 were well documented (eg, 6399 full-time equivalent nurses and 1906 doctors [13]), no data were gathered on nurse staffing, skill or grade mix, or the quality of the nurse work environment [10], all of which would be highly significant to quality improvement strategies.

Higher nurse staffing is positively associated with patient safety and nurse outcomes in general hospitals [12]. This positive association between nurse staffing and patient safety is also known, but less studied, in psychiatric settings [16,17]. In addition to nurse staffing, work environment factors, including nurses’ perceived workload, relationship with physicians, and leadership, are reported as structural factors that influence patient and nurse outcomes in psychiatric hospitals [18].

In addition, to assess nursing processes, rationing of care, that is, the partial or complete omission of care because of a lack of resources [19,20], has frequently been observed [19]. Higher proportions of rationing care are associated with lower staffing levels [21]. To date, the rationing of care has not been measured in psychiatric hospitals [20].

### Patient Characteristics and Outcomes in Swiss Psychiatric Hospitals

In 2017, the most frequent diagnoses at admission to Swiss psychiatric hospitals were affective disorders (32.1%), schizophrenia (16.4%), and anxiety or dissociative disorders (13%) [13]. Of all the admissions, 19.7% were involuntary [22]. Involuntary admissions are only allowed if the treatment is absolutely indispensable, for example, in cases where patients pose a threat to themselves or others, and care cannot be provided in any other form, such as outpatient clinics [22]. In psychiatric inpatient facilities, the risk of patient violence is higher than that in other care settings [23]. Although this is mainly because of the acuity of patients’ psychiatric symptoms, it also relates to the curtailment of patients’ personal freedom in inpatient settings [24,25].

Patient outcome data were mandatorily collected for the Swiss National Association for Quality Development in Hospitals and Clinics. It included clinician-rated symptom burdens and self-rated symptom burdens at admission and discharge, as well as patient satisfaction and any coercive measures taken (ie, seclusion, restraints and coercive medication [26]). Aggregated and publicly reported at the hospital level, these patient outcomes serve as benchmarks for psychiatric institutions.

This purpose of this study is to describe the structure, processes, and nurse and patient outcomes in Swiss psychiatric hospitals. The results will deepen our understanding of the quality of care in psychiatric inpatient settings.

### Aims

This study aims to (1) describe the structures and processes of nursing care, (2) describe patient outcomes at the unit level, and (3) explore possible associations between the nursing work environment and patient outcomes in psychiatric hospitals.

### Framework

**Figure 1:**
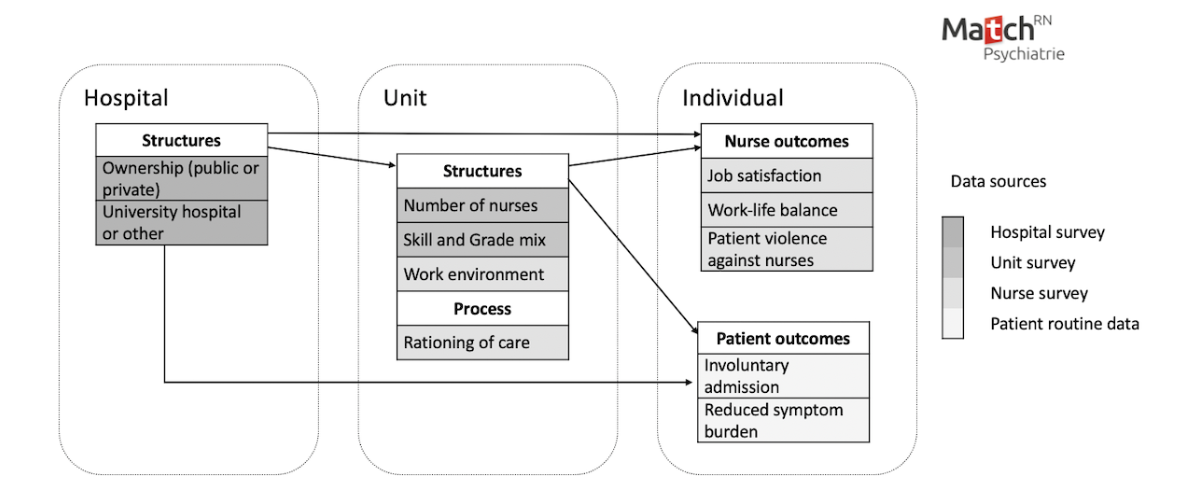
The framework of Match^RN^ Psychiatry.

The Match^RN^ Psychiatry framework deals with critical information collected at the hospital, unit, and individual levels (Figure 1). As a framework, it was adapted from the Match^RN^ study, which was conducted for acute care hospitals [27], and was originally based both on the Donabedian SPO model [6] and Donaldson contingency theory [28].

According to Donabedian [6], quality of care can be evaluated in terms of structure (health care setting characteristics), processes (clinical processes in health care settings), and outcomes (eg, patient status after application of the process). The three SPO dimensions are interlinked, where structures provide the basis for the provision of a process that affects outcomes. Donaldson contingency theory assumes that organizations fit into their environment; for example, units fit into the hospital, and hospitals fit into the health care system. To achieve an appropriate fit, the organization must adapt to its environment [28]. A health care organization’s fit is characterized by its performance, for example, quality of care (nurse and patient outcomes) and the efficiency of its services, for example, structure and process [29]. Combining the 2 models allows the mapping of the Donabedian quality criteria while highlighting interdependent and organizational dynamics at the hospital, unit, and individual (nurse and patient) levels (Figure 1).

## Methods

### Design

Match^RN^ Psychiatry is a cross-sectional multicenter study of Swiss German psychiatric hospitals.

### Setting and Sample

This study included psychiatric hospitals with units for adult inpatient care in the German-speaking part of Switzerland. All 40 institutions with membership in the Swiss Psychiatric Nursing Leaders Association (Vereinigung Pflegekader Psychiatrie Schweiz, 40/50, 80% of all Swiss psychiatric hospitals) were invited to participate. A total of 13 psychiatric hospitals decided to enroll in the study, leading to a convenience sample at the hospital level.

All units for adult inpatients were eligible; however, the hospitals decided which units to enroll, resulting in a convenience sample (units per hospital: range 3-17).

This study sample consisted of 115 inpatient units, including their nursing workforce, totaling approximately 1300 registered nurses and health care assistants. In addition, routine data from all inpatient cases treated in these units in 2019 will be included. Other inpatient areas, such as forensic units, were excluded.

### Variables and Measurement

The Match^RN^ Psychiatry variables and measurements include patient routine data and survey data at the hospital, unit, and individual nurses level based on the Match^RN^ acute care survey [27].

### Hospital Survey

The hospital-level survey asks for hospital characteristics: ownership status (private or public), hospital type (university or nonuniversity hospital), and hospital size (number of beds).

### Unit Survey

On the basis of the Match^RN^ acute care survey [27], the 57-item unit-level survey assesses each unit’s staffing (eg, number of nurses and use of agency nurses), staff planning principles (eg, skill mix, nurse-to-patient ratio), and influence of the COVID-19 pandemic on the unit (Table 1). The unit managers will be asked to complete it.

**Table 1:** Variables and measurements in the units’ survey.

Topic			Description		Measurements
Unit characteristics			5 items assessing the name, specialization, number of beds, average length of stay, and bed occupancy at the unit		2 text items, 3 number items
**Workforce**					
	Numbers of FTEs^a^	2 items from the Match^RN^ study [27] assessing the FTE of nurses according to educational background and function		Number of FTEs	
	Agency nurses	7 items from the Match^RN^ study [27] assessing frequency, duration, and attitude for agency nurses’ use		4 items: 10-point Likert-type scale from 1 (strongly disagree) to 3 (strongly agree); 3 items: 6-point Likert-type scale from 1 (never) to 6 (several times a week)	
**Organization of nurse service**					
	Resources allocation	20 items from the Match^RN^ study [27] assessing resources allocation at the unit		4 items: 10-point Likert-type scale from 1 (very low) to 3 (very high); 16 items with various multiple answer options	
	Work schedule	16 items from the Match^RN^ study [27] assessing the responsibility, influence, and flexibility in work schedules of nurses at the unit		4 items: 10-point Likert-type scale from 1 (strongly disagree) to 3 (strongly agree); 12 items with various multiple answer options	
**Influence of COVID-19 at the unit**					
	COVID-19 at the unit	4 investigator-developed items assessing whether patients with COVID-19 are at the unit; teaching and use of personal protection equipment		3 items: 2 answer options (yes or no); 1 open-text item	
**Career characteristics of the unit manager**					
	Career characteristics	5 items from the Match^RN^ study [27] assessing qualification level, years in nursing, years in psychiatric care		N/A^b^	

^a^FTE: full-time equivalent.

^b^N/A: not applicable.

### Nurse Survey

On the basis of the Match^RN^ acute care survey [27], the 164-item Match^RN^ Psychiatry nurse survey captures variables of the psychiatric setting (Multimedia Appendix 1 [27,30-42]).

This survey fulfills 2 main objectives. First, it collects data on structural factors, such as the number of nurses present on the last shift, quality of the nurse work environment (measured via a modified version of the Practice Environment Scale of the Nursing Work Index [30]), and safety culture (measured via the Safety Attitude Questionnaire [31]). Second, it asks about work processes in the unit, including, for example, a version of the rationing of care [19] developed and modified by the Match^RN^ Psychiatry study team that is fit for use in psychiatric inpatient settings. The modification process included a literature review and pilot test with experts from inpatient settings in psychiatric hospitals. In addition, it includes items on nurse outcomes such as job satisfaction, well-being, and experiences with patient violence against nurses [32], as well as on sociodemographics (eg, age and gender), and professional experience in nursing (eg, years in nursing and years in psychiatric care).

### Patient Data

We will use 17 items from the Swiss National Association for Quality Development in Hospitals and Clinics questionnaire, which is mandatory for all psychiatric hospitals. This includes data on all inpatients who were hospitalized in participating units during 2019 (Table 2). In addition to demographic details (age and sex), clinical data will be included (medical diagnoses [with International Classification of Diseases-10 codes] and the reduction of symptom burden), along with symptom burden data taken at admission and discharge via the Health of the Nation Outcome Scale (HoNOS) [43].

**Table 2:** Variables and measurements for patient routine data.

Topic and variable				Measurement
**Demographics**				
	Age			In years at admission
	Gender			Female or male
**Clinical data**				
	**Length of stay**			
		1 item calculated from the date of admission and date of discharge	Number of days	
	**Medical diagnosis**			
		ICD-10^a^ code	5-digit code	
	**Symptom burden**			
		All 12 items of the HoNOS^b^ [44] Overactive, aggressive, disruptive, or agitated behavior Nonaccidental self-injury Problem drinking or drug taking Cognitive problems Physical illness or disability problems Problems with hallucinations and delusions Problems with depressed mood Other mental and behavioral problems Problems with relationships Problems with activities of daily living Problems with living conditions Problems with occupation and activities	5-point Likert-type scale from 0 (no problem) to 4 (severe or very severe problem) measured at admission and discharge	

^a^ICD-10: International Classification of Diseases-10.

^b^HoNOS: Health of the Nation Outcome Scale.

The HoNOS, which is to be completed by the responsible health professional, includes 12 items, such as *overactive, aggressive, disruptive, or agitated behavior*, *nonaccidental self-injury*, and *problems with activities of daily living*. The German-language version of the HoNOS showed satisfactory results for feasibility (range of missing values 1.3%-4.5% for 11 items) and satisfactory retest reliability (interclass correlation 0.80-0.91, for 9 items) [44]. Coercive measures will be assessed for each patient case using the number and duration of seclusion, restraint, and coercive medication occurrences, as well as admission status (ie, involuntary or voluntary).

### Validity and Reliability

Except for the modified version of the rationing of care scales and the questions about COVID-19 in the unit survey, all data collection instruments have been tested for validity and reliability in previous international and national studies [33,34,45].

We used established or pretested German-language versions for the nurse survey to ensure the validity and reliability of the study instruments. In addition, the nurse survey items were pilot tested for content validity and comprehensibility in a group of 29 nurses from 5 psychiatric hospitals.

### Data Collection

Data collection at the hospital, unit, and individual levels (nurses and patients) was initially planned for September 2019 to April 2020. However, because of the COVID-19 pandemic, only the nurse survey was completed within this period. Unit- and hospital-level data collection was completed in June 2021.

Each hospital can choose whether data are collected via a web-based survey or a paper-pencil questionnaire. At each participating hospital choosing the latter, a single point-of-contact person will be responsible for the onsite organization of the questionnaire distribution. Individual study participation is entirely voluntary. Informed consent will be obtained by filling out and submitting the questionnaire. The participating hospitals will provide patient routine data at the unit level. As these data include no information that could be used to identify individual patients or nurses, anonymity will be guaranteed. Data at all levels will be collected once.

### Data Analysis

After checking the data quality for plausibility and missing data, we will conduct descriptive analyses for all variables using frequencies and percentages for categorical variables, with means and SDs reported for continuous variables. We will assess the dimensionality of the rationing of care items using a Mokken scale analysis [46]. To explore the relationship between nurse staffing, including the patient to nurse ratio and work environment as exposure variables and patient’s symptom burden or nurse outcomes such as work-life balance, we will use linear mixed models for normally distributed data and generalized linear mixed models for dichotomous outcomes. For the latter, we will calculate odds ratios and 95% CIs. For example, to assess work-life balance, a generic model would have the following structure:


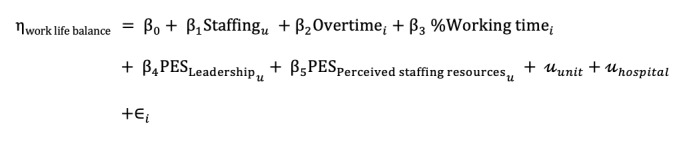


where the outcome work-life balance is a normally distributed outcome, which is predicted by unit-level variables (eg, staffing and leadership), individual-level variables (eg, age, working time, and family status), and random intercepts for unit and hospital ID.

All statistical analyses will be performed using the software R (R Foundation for Statistical Computing), version 4.X for MacOS [47]. To minimize confounder bias and determine the robustness of the effects, we will conduct sensitivity analyses for all inferential regression analyses [48].

### Ethical Considerations

The responsible ethics committee (Ethics Commission Northwest and Central Switzerland) ruled the status of the Match^RN^ Psychiatry as an exempt (project ID: Req-2019-00589).

The data collection procedure was approved by the data protection officer of the University of Basel.

The nurse questionnaire will be distributed with a cover letter explaining the study’s purpose and data protection measures, assuring confidentiality and anonymity, and emphasizing that participation is voluntary.

Data protection and confidentiality will be ensured by using codes for each psychiatric hospital and unit so that only the research team at the University of Basel’s Institute of Nursing Science will be able to identify study sites and units. Each individual nurse respondent will remain anonymous. The patient outcome routine data will be provided anonymously from participating psychiatric hospitals. The anonymized data will be deposited in the Zenodo open-access research data repository.

### Dissemination of Findings

First, benchmark reports, including unit-level results, will be provided to the participating institutions, allowing the comparison of findings and interhospital learning. A national report with key descriptive results will be published, providing nonparticipating psychiatric hospitals access to the findings. To further support psychiatric hospitals, a congress will be held to promote and discuss the results with and between them. Furthermore, the study results will be communicated to the study sites on demand. We also envision the publication of study results via scientific journals and scientific conferences.

## Results

The response rate from the nurse survey was 71.49% (1209/1691). All data are currently being checked for consistency and plausibility. The Match^RN^ Psychiatry study is funded by the participating psychiatric hospitals and the Swiss Psychiatric Nursing Leaders Association (Vereinigung Pflegekader Psychiatrie Schweiz).

## Discussion

For the first time, the Match^RN^ Psychiatry study will assess the quality of care in Swiss psychiatric hospitals by considering all relevant structures, processes, and patient and nurse outcomes. On the basis of the relationships indicated between these variables, they can later be targeted to maintain or improve the quality of care in Swiss psychiatric hospitals in accordance with global initiatives, including the World Health Organization’s Quality Rights Initiative [7,8,49,50]. The participating psychiatric hospitals will benefit from the planning and regulation of nurse staffing. By improving Swiss psychiatric hospitals’ understanding of their nurse work environment factors’ relationships with specific patient outcomes, Match^RN^ Psychiatry will allow and encourage Swiss psychiatric hospitals to target interventions that will improve both nurse and patient outcomes. Future research should also provide a foundation for cantonal, national, and international studies and comparisons.

## Appendix

Multimedia Appendix 1 Variables and measurements in the nurses’ survey.

## Abbreviations

HoNOS: Health of the Nation Outcome Scale
SPO: structure-process-outcome
