# Supplementary material for: Matching Registered Nurse Services With Changing Care Demands in Psychiatric Hospitals: Protocol for a Multicenter Observational Study (MatchRN Psychiatry Study)
Source: JMIR Res Protoc. 2021 Aug 17;10(8):e26700. doi: 10.2196/26700 (PMC8408752; doi:10.2196/26700)
Supplement: Multimedia Appendix 1 [file resprot_v10i8e26700_app1.docx]

Variables and measurements in the nurses’ survey

**Table S1.** Variables and measurements in the nurses’ survey.

| Topic | | Description | Measurements |
| --- | --- | --- | --- |
| **Work environment** | | | |
|  | Staffing and resources adequacy | - 4-item subscale of the revised PES-NWI^a^ [30] assessing whether enough time was provided to discuss patient care problems as well as enough RN^b^ staff and support service available to do all necessary work well | - 4-point Likert-type scale from 1 (strongly disagree) to 4 (strongly agree) |
|  | Nurse manager ability, leadership, and support of nurses | - 4-item subscale of the revised PES-NWI [30] assessing support by direct supervision, leadership ability of the head nurse, and recognition for jobs well done | - 4-point Likert-type scale from 1 (strongly disagree) to 4 (strongly agree) |
|  | Nursing foundations for quality of care | - 9-item subscale of the revised PES-NWI [30] assessing whether a quality assurance program was active on the unit, as well as a preceptor program for new RNs; nursing care based on a nursing model and a clear philosophy of nursing; patient care assignments that foster continuity of care; and high expected standards of nursing care, including working with competent nurses | - 4-point Likert-type scale from 1 (strongly disagree) to 4 (strongly agree) |
|  | Collegial nurse-physician relations | - 3-item subscale of the revised PES-NWI [30] assessing working relationships, teamwork, functional collaboration; in addition, investigator-developed nurse relationships with psychologists (2 items), social-workers (2 items), and medical therapeutic services (2 items) | - 4-point Likert-type scale from 1 (strongly disagree) to 4 (strongly agree) |
| **Job satisfaction** | | | |
|  | General job satisfaction | - Single item of the RN4CAST^c^ study [33] assessing satisfaction with the current job | - 4-point Likert-type scale from 1 (very dissatisfied) to 4 (very satisfied) |
|  | Intention to leave | - 3 items of the NEXT^d^ study [35] assessing thoughts of quitting job during the last 12 months and leaving the job setting or the profession | - 5-point Likert-type scale from 1 (never) to 5 (every day) |
|  | Recommendation of hospital | - 2 items of the RN4CAST study [33] assessing recommendation of hospital as employer or for treatment | - 4-point Likert-type scale from 1 (definitely no) to 4 (definitely yes) |
| **Health status** | | | |
|  | Energy and well-being | - 5-item subscale of the COPSOQ^e^ [32] assessing physical exhaustion, emotional exhaustion, feeling worn out, going to work when feeling sick, inability to forget work in leisure | - 5-point Likert-type scale from 1 (always) to 5 (never) |
|  | Work-life balance | - 8-item Work-life climate scale [36] assessing frequencies during the past week of skipping a meal, eating a poorly balanced meal, working through a shift without any breaks, arriving home later than planned, having difficulty sleeping, sleeping <5 hours in a night, changing personal or family plans because of work, and feeling frustrated by technology | - 4-point Likert-type scale from 1 (always; 5-7 days/week) to 4 (never; 0 day/week) |
| **Quality and safety** | | | |
|  | General quality of nursing care and patient safety | - 5 items of the RN4CAST study [33] assessing general quality of nursing care and patient safety on the unit and changes in the last year | - 5-point Likert-type scale from 1 (not enough) to 5 (excellent) |
|  | Safety and teamwork climate | - 13-item Safety and teamwork climate subscale of the SAQ^f^ [31] assessing, for example, how easy it is to ask questions and to speak about suggestions, problems and mistakes, interdisciplinary collaboration, and the feedback and support of the team as well as how easy it is to resolve disagreements | - 5-point Likert-type scale from 1 (strongly disagree) to 5 (strongly agree) |
| **Work schedule** | | | |
|  | Work schedule last week | - 2-item Work schedule of last week from Match^RN^ study [27] assessing the previous week’s planned work schedule and overtime | - For 1 week for each day start and end of shift, overtime in minutes |
|  | Influence on schedule | - 13-item Influence on schedule scale from Match^RN^ study [27] assessing possibilities to change shifts, influence schedule planning | - Various Likert-type scales, for example, from 1 (no changes are possible) to 3 (changes are rather easy) or 5 items: 5-point Likert-type scale from 1 (never) to 5 (always) |
| Last shift | | - 7-item Last shift from Match^RN^ study [27] assessing start and duration of last shift, number of patients on the unit, staffing; in addition, 5 items (investigator-developed) assessing the number of patients with aggressive behavior, suicidality, somatic diagnosis, hourly monitoring, number of admissions or discharges | - 1 item: 3 answer options (day shift, afternoon or evening shift, night shift); 1 item: 2 answer options (yes, no); 3 items: time variable; 9 items: number |
| **Subjective workload** | | | |
|  | Overall subjective workload | - 7-item overall subjective workload subscale from NASA-TLX^g^ [37] assessing mental, physical, emotional, and temporal demand, along with frustration, effort, and performance - 7-item subjective workload subscale from NASA-TLX [37] assessing the previous shift’s mental, physical, emotional, and temporal demand, along with frustration, effort, and performance | - 7-point Likert-type scale from 1 (low) to 7 (high) - 20-step bipolar scales from low to high |
| **Rationing of care** | | | |
|  | Rationing of direct care | - 13-item Rationing of direct care subscale from BERNCA^h^ [38] adapted to psychiatric setting, assessing whether interventions were performed with the patient as planned | - 5-point Likert-type scale from 1 (never) to 5 (always), 2 additional answer options (intervention is not in my area of responsibilities, intervention does not occur on the unit) |
|  | Rationing of indirect care | - 6-item Rationing of indirect care subscale from BERNCA [38] adapted to psychiatric setting, assessing whether interventions without the patient were performed as planned | - 5-point Likert-type scale from 1 (never) to 5 (always), 2 additional answer options (intervention is not in my area of responsibilities, intervention does not occur on the unit) |
|  | Rationing of practice development | - 2-item investigator-developed Rationing of practice development subscale assessing whether interventions were performed on the unit for practice development | - 5-point Likert-type scale from 1 (never) to 5 (always), 2 additional answer options (intervention is not in my area of responsibilities, intervention does not occur on the unit) |
|  | Rationing of care in last shift | - 1-item Rationing of care in last shift subscale from Match^RN^ study [27] assessing the percentage of rationing of care on last shift | - Percentage 0-100 |
| **Patient violence against nurses** | | | |
|  | Nurses’ experience of patient violence during the last month | - 5 investigator-developed items based on the Perception of Prevalence of Aggression Scale [39] assessing the previous month’s frequencies of verbal violence, physical violence, and sexually harassing violence | - 6-point Likert-type scale from 1 (never) to 6 (daily) |
|  | Nurses’ experience of patient violence during career | - 2 investigator-developed items based on the Perception of Prevalence of Aggression Scale [39], assessing the frequency of physical assault and sexual assault during overall work in psychiatric care | - 3-point Likert-type scale from 1 (never) to 3 (3 times or more) |
|  | Nurse attitudes toward patient violence | - 3 investigator-developed items assessing whether being verbally threatened, physically attacked, or sexually harassed are part of work in psychiatric care | - 11-point Likert-type scale from 0 (strongly disagree) to 3 (strongly agree) |
|  | Nurses level of self-efficacy in dealing with patient violence | - 13 investigator-developed items: nature of questions and scales based on the Bandura Guide for constructing perceived self-efficacy scales [40] assessing how confident nurses feel dealing with patients who verbally threaten, physically attack, or sexually harass them | - 11-point Likert-type scale from 0 (very unconfident) to 3 (very confident) |
|  | Participation in courses focused on patient violence | - 3 investigator-developed items based on the Leo Regeer method [41] assessing whether nurses participated in courses focused on patient violence | - 1 item: 2 answer options (yes, no); 1 item: 3 answer options (never, during the last 12 months, more than 12 months ago); 1 item: 4-point Likert-type scale from 1 (never) to 4 (always) |
| **Absenteeism and presenteeism** | | | |
|  | Absenteeism | - Single item from Swiss Health Survey [42] | - Frequency |
|  | Presenteeism | - Single item of SHURP^i^ study [34] | - Frequency |
| **Career characteristics and demographics** | | | |
|  | Career characteristics | - 6 items assessing qualification level, years in nursing, years in psychiatric care | - N/A^J^ |
|  | Demographics | - 2 items assessing age and gender | - Number, 2 answer options (female, male) |

^a^PES-NWI: Practice Environment Scale of the Nursing Work Index.

^b^RN: registered nurse.

^c^RN4CAST: Nurse Forecasting: Human Resources Planning in Nursing.

^d^NEXT: Nurses’ Early Exit Study.

^e^COPSOQ: Copenhagen Psychosocial Questionnaire.

^f^SAQ: Safety Attitude Questionnaire.

^g^NASA-TLX: National Aeronautics and Space Administration-Task Load Index.

^h^BERNCA: Basel Extent of Rationing of Nursing Care.

^i^SHURP: Swiss Nursing Homes Human Resources Project.

^j^N/A: not applicable.
